# Toxicities effects of pharmaceutical, olive mill and textile wastewaters before and after degradation by *Pseudomonas putida *mt-2

**DOI:** 10.1186/1475-2867-12-4

**Published:** 2012-02-07

**Authors:** Hedi Ben Mansour, Afef Dellai, Yosra Ayed

**Affiliations:** 1 Laboratoire de biotechnologie et Valorisation de Bio Géo Ressources (LBVBGR) Institut Supérieur de Biotechnologie - ISBST BioTechPole Sidi Thabet Université Manouba, Ariana 2020, Tunisie; 2 Laboratoire de Recherche des Substances Biologiquement Compatibles, Faculté de Médecine Dentaire, Rue Avicenne, Monastir 5000, Tunisie

**Keywords:** Industrial wastewater, Bioremediation, *Pseudomonas putida *mt-2, Algogenic effect, Convulsant effect

## Abstract

**Background:**

Removal of numerous classes of chemical pollutants from the industrial wastewater such as textile, pharmaceutical and olive mill using conventional wastewater treatment, is incomplete and several studies suggested that improvement of this situation would require the application of biological treatment techniques. Dyes, polyphenols and drugs are an environmental pollutants extremely toxics to plants and other living organisms including humans. These effluents were previously treated by *Pseudomonas putida*. The main of this work was to evaluate the *in vivo *toxicity of the three wastewaters.

**Methods:**

Writhes and convulsant effect of effluents were carried out and were compared to the treated effluents. Only pharmaceutical wastewater was exhibited a convulsant effect which observed in mice treated by effluent. On the other hand, all industrial wastewater induced significantly an algogenic effects particularly when mice were treated by the pharmaceutical wastewater (Number of writhes = 44).

**Conclusion:**

Toxicity was totally removed when mice were treated by the bio remediated effluent. This indicates that *P. putida *was able to completely detoxify the toxic industrial effluent.

## Background

Over the last century, continued population growth and industrialization have resulted in the degradation of various ecosystems on which human life relies on. In the case of ocean and river quality, such pollution is primarily caused by the discharge of inadequately treated industrial wastewater. Due to increasing awareness about the environment and more stringent environmental regulations, treatment of industrial wastewater has always been a key aspect of research. Much work has been done in developing and testing newer techniques and their combinations for wastewater treatment [1,2]. In Tunisia, some of the industrial sectors presenting a serious threat to the surrounding eco-system due to its usage of a vast array of organic compounds which can therefore reach natural water resources are the textile, pharmaceutical and olive mill industries. The quality of these industrial effluents, thus, has substantial influence on the quality of surface water. Raw wastewaters are highly loaded with organic matter up to several grams per liter of organic carbon that may consist of well- and poorly degradable bio-genic and synthetic organic compounds (such as dyes, polyphenols, pharmaceutical drugs, antibiotic, heavy metals etc.) [3-5]. Pharmaceutical, olive mill and textile industrial effluents require treatment before delivery to municipal treatment plants or the direct discharge into surface waters. In the treatment of these wastewaters, biological treatment appears to be a promising technology compared to physicochemical treatment methods. These least, usually developed by industries, are frequently regarded as a regulatory obligation, increasing capital and running costs and yielding negative economic returns.

In previous work [3-5]*P. putida*, cultivated under continuous shaking incubation, is revealed able to treat these industrial effluents. This result is very important because the same studies indicated that TW, PW and OMW exhibited highly *in vivo *and *in vitro *genotoxicity, lipoperoxidation and inhibit strongly cholinesterase activity.

In this study, we tested the ability of the TW, PW and OMW before and after treatment with *P. putida *mt-2 to induce algogenic and convulsant effects in mice.

## Methods

### Chemicals

Pentylenetetrazole (PTZ), acetic acid and phenobenzoquinone (PBQ) were purchased from Sigma-Aldrich (Steinheim, Germany).

### Wastewaters

Toxicity assessment experiments were performed with three industrial wastewaters (i) The textile wastewater used in the present study was obtained from an textile ennoblement industry (located in Sousse: Center of Tunisia), this effluent contains five azo dyes Yellow GR (≈100 mg/l); Red FE3B (≈50 mg/l); Blue BRR (≈50 mg/l); Blue GLL (75 mg/l) and Brown BL (75 mg/l); dispersant (sarabide 10 g/l) and two fixative agents (sodium sulphate 20 g/l and Rewin ACP 5 g/l), finally potassium nitrate (1 g/l), and some heavy metals (Zn, Pb, Cu, Fe) [3] (ii) The Olive mill wastewater (OMW) was obtained from an olive oil production plant (located in Melloulech: centre of Tunisia), the OMW was derived from discontinuous process for extraction of olive oil (chemical characteristics: pH: 5.1; COD: 93 g/l; N: 1340 mg/l; P: 720 mg/l; K: 6200 mg/l; phenols: 8400 mg/l; glucose: 1200 mg/l) [4] and finally (iii) Mixture of two pharmaceutical wastewater obtained from two pharmaceutical industries located in the north and center of Tunisia. The wastewater contained a mixture of organic compounds among which were celiprol, losartan, enalapril, buflomedil, losartan and carvedilol (cardiology drug); oseltamivir (anti-viral drug); sucralose and simvastatine (nutrition metabolism drug) and finally ciprofloxacin (antibiotic drug) [5].

### Wastewaters bioremediation

Biodegradation was conducted with *P. putida *mt-2 under aerobic condition. The biodegradation conditions were previously described [3-5].

### Animals

Swiss mice (20-30 g) of both sexes, provided from Pasteur institute (Tunis, Tunisia) were used. Animals were fed a standard diet ad libitum and allowed free access to drinking water. Housing conditions and *in vivo *experiments were approved according to the guidelines established by the European Union on Animal care (CEE Council 86/609).

### Convulsant and writhing studies in mice

Animals were randomly divided into nine groups:

1. Animals given a single dose (10 mL/kg bw) of culture medium run in the presence of *P. putida *mt-2 and without textile effluent as negative control group.

2. Animals given a single dose of pentylenetetrazole (PTZ) (0.9 g/Kg w) which constitute the positive control group for the convulsant test.

3. Animals given a single dose of PBQ (0.4 g/Kg w) which constitute the positive control group for the Writhing test.

4. Animals given a single dose of untreated TW, administrated intraperitoneally (ip) (10 mL/kg bw).

5. Animals given a single dose of untreated PW, administrated intraperitoneally (ip) (10 mL/kg bw).

6. Animals given a single dose of untreated OMW, administrated intraperitoneally (ip) (10 mL/kg bw).

7. Animals given a single dose of treated TW by *P. putida *mt-2, administrated intraperitoneally (ip) (10 mL/kg bw).

8. Animals given a single dose of treated PW by *P. putida *mt-2, administrated intraperitoneally (ip) (10 mL/kg bw).

9. Animals given a single dose of treated OMW by *P. putida *mt-2, administrated intraperitoneally (ip) (10 mL/kg bw).

It is of note that our results clearly showed that the selected dose of wastewaters and its biodegradation derivatives administrated alone to animals did not exhibited change of the rate of mortality, the body weight, the feed intake and the size and shape of liver and kidney.

For the convulsion study, the time taken before the onset of colonic convulsions and mortality were recorded. These parameters were compared in treated animals, by each effluent, with those of control animals (PTZ) (0.9 g/Kg w), in order to assess the convulsant effect.

For the writhing test the number of writhing was recorded during 30 min commencing 5 min after the intraperitoneal injection. A writhe is indicated by abdominal constriction and stretching of at least one hind limb according to the method described by Siegmund et al. [6]. Results were compared to the control animals (PBQ 0.4 g/Kg w).

### Statistical analysis

Data are presented as the mean ± standard error (s.e.m). Statistical analysis was performed using Student's *t*-test. The significance of difference was considered to include values of *P *< 0.05.

## Results

It is of note that our results clearly showed that the selected effluents (TW, OMW and PW) and their biodegradation derivatives administrated alone to animals did not have any toxic effect (mortality, body weight, feed intake).

Results showed that no convulsion effects were shown by the tested TW and OMW (Table 1). The time taken before the onset of colonic convulsions and mortality were 0% respectively which was compared to the negative control (distillated water). However, PW wastewater exhibited a high power to induce a convulsant behaviour which observed in mice; in fact the time taken before the onset of colonic convulsions and mortality was 17 ± 1.3 which was comparable to the positive control PTZ 90 mg/kg bw (15 ± 1.16). Onset of seizure observed in mice treated by PW (230.5 ± 13.5) exceeds that of PTZ (180.16 ± 33.66).

**Table 1:** convulsant effect of the intraperitoneal administration of untreated and treated industrial TW, OMW and PW in comparison with the pentylenetetrazole (PTZ) (90 mg/ml) in mice

Treatment	Onset of seizure	Duration (s)	Mortality (%)
PC	180.16 ± 33.66**	15 ± 1.16*	100

NG	0	0	0

Untreated TW	0	0	0

Untreated OMW	0	0	0

Untreated PW	230.5 ± 13.5**	17 ± 1.3*	0

treated TW	0	0	0

treated OMW	0	0	0

treated PW	0	0	0

Positive control: pentylenetetrazole (PTZ) (90 mg/ml); Negative control: saline 10 ml/kg

TW: Textile wastewater; OMW: olive Mill wastewater and PW: pharmaceutical wastewater

Values are expressed as mean ± s.e.m. *P < 0.01; *P < 0.001 n = 6 animals

Table 2 showed that all tested effluent (TW, OMW and PW) showed a significant effect to induce writhes. However, PW induced writhing more than TW and OMW, in fact, the number of writhing was recorded during 30 min were 44 ± 2; 23 ± 5 and 26 ± 3, respectively. Pharmaceutical wastewater was more toxic than PBQ (reference compound). Writhing effect was completely disappears in mice treated by the tested effluent treated and the number of writhes which are comparable to those observed in mice treated by distillated water (Table 2).

**Table 2:** Number of writhes in mice treated with each industrial wastewater before and after remediation with *P.putida *mt-2

Tested compound	Number of writhes
	**± s.e.m**.
NC	00 ± 00

PC	39 ± 2**

Untreated TW	23 ± 5 *

Untreated OMW	26 ± 3*

Untreated PW	44 ± 2**

Treated TW	00 ± 00

Treated OMW	3 ± 1

Treated PW	00 ± 00

Positive control: phenobenzoquinone (PBQ); Negative control: distillated water

TW: Textile wastewater; OMW: olive Mill wastewater and PW: pharmaceutical wastewater

Values are expressed as mean ± s.e.m. *P < 0.01; **P < 0.001 n = 6 animals

## Discussion

A common practice in Tunisia is to discharge untreated industrial effluents directly into neighbouring water bodies or onto agricultural land. As a result the quality of some local streams and rivers has been degraded to the point where the water is not safe for human or livestock use or for irrigation. Textile, olive oil mill and pharmaceutical wastewaters (TW, OMW and PW) are an environmental pollutants extremely toxics to plants and other living organisms including humans. In previous work [3-5]*Pseudomonas putida *mt-2 was revealed able to treat these effluents. However, we cannot accurately locate the toxicity of effluents and a key question arises is that such treatment leads to detoxification. The level of toxicity in the DNA, previously described [3-5], may not be sufficient and does not reflect the toxicity of the other part of the body among other things the brain. In this work, a single dose of intraperitoneal administration of pharmaceutical wastewaters caused strongly clonic convulsions (230.5 onset of seizure) which are higher than the reference drug PTZ (180.16 onset of seizure). Despite the difference in chemical structure between PTZ and drugs of PW contents, the latter has a higher convulsion effect. This could be explaining by several hypotheses. In fact, synergic effects between some or all drug of PW contents are responsible for the convulsion effects, or, metabolites deriving from UV or VIS degradation of these drug when released into the environment could show a similar chemical structure than PTZ, and consequently are responsible for the observed convulsion effect. Then, PW could induce epilepsy; this later is one of the most common serious neurological conditions. According to Aalbers et al. [7] and Luisa [8] the forbrain is involved in the expression of clonic seizures, whereas the activation of brainstem structures participates in the expression of the tonic component. Convulsive seizures could be attributed to the presence of drugs in PW which blocked γ-aminobutyric acid (GABA) receptor Cl^- ^channels [7,8]. However, the two other effluents, TW and OMW, do show any convulsing effect. Seizures have traditionally been recognized as a symptom of abnormal neuronal synchronization, and until recently have been thought to be a result of aberrant synaptic communication [9,10]. This idea was confirmed by other models such as visceral pain. Among the several models of visceral pain, writhing test has been mostly used as a standard screening method [11]. All tested wastewaters are reveled able to induce algogenic effects. In fact intraperitoneal administration of the three industrial wastewaters produces significant abdominal contractions throughout the entire period of observation. Similarly, the PW has been proven to be very active and strongly induces pain that result in a remarkable number of cramps (44 ± 2), well above the PBQ (drug reference) (39 ± 2). TW and OMW also induce effects algogenic but lesser degree. The algogenic effect could be attributed especially to the presence of pharmaceutical drug, dyes and phenolic compounds in the PW, TW and OMW, respectively.

The induction mechanism of algogenic effects by these products could be defined according to two hypotheses. Molecules contained in effluents acts indirectly by inducing the release of endogenous mediator, which stimulates the nociceptive neurons sensitive to NSAIDs (non-steroidal anti-inflammatory drugs) and/or opioids [12] but these molecules-induced writhing response could believed to be produced by the liberation of endogenous substance(s), notably metabolites of the arachidonic cascade [12,13].

According to the toxic effects observed in these effluents, the search of a treatment is imperative. However, removal of numerous classes of pharmaceuticals, textile and olive mill from the industrial wastewater, using conventional wastewater treatment, is incomplete. In this work we suggested that improvement of this situation would require the application P. putida to the treatment of these effluents. This is particularly important for the treatment of industrial effluents, released from pharmaceutical, textile and olive mill industries, which can contain rather high concentrations of toxic compounds. In fact, any toxicity was observed when tested effluents treated by *P. putida *mt-2.

## Competing interests

The authors declare that they have no competing interests.

## Authors' contributions

HBM: Was responsible for the conception and design, testing and data acquisition, analysis and data interpretation and drafted the manuscript. AD: made contribution to the study algogenic activities. YA: made contribution to convulsing activity. All authors read and approved the final manuscript.
